# Classification of temporomandibular joint internal derangement based on magnetic resonance imaging and clinical findings of 435 patients contributing to a nonsurgical treatment protocol

**DOI:** 10.1038/s41598-021-00456-7

**Published:** 2021-10-22

**Authors:** Ayman F. Hegab, Hossam IAbd Al Hameed, Khaled Said Karam

**Affiliations:** 1 Department of Oral and Maxillofacial Surgery, Faculty of Dental Medicine, Al Azhar University, Cairo, Egypt; 2 Faculty of Medicine for Men, Al Azher University, Cairo, Egypt

**Keywords:** Clinical trial design, Oral diseases

## Abstract

This prospective clinical study aimed to establish a new classification system for TMJ internal derangement based on MRI in correlation with clinical findings contributing to a nonsurgical treatment protocol. A consecutive sample of 435 internal derangement patients was enrolled in the study. Clinical and MRI studies were used to establish the new classification system. A total of 747 joints were classified according to our staging system and received treatment according to the associated nonsurgical treatment protocol. The primary outcome variables were maximum voluntary mouth opening and visual analogue scale pain scores. The secondary outcome variable was joint sound. Statistical analysis of the differences between pretreatment and posttreatment measurements showed an increase in mouth opening throughout the study period (*P* < 0.001 at 12 m posttreatment). Statistical analysis of the **VAS** scores showed a statistically significant decrease in all study groups during all study periods, with *P* < 0.0001 at 12 months posttreatment. Statistical analysis of **joint sounds** showed significant improvement during all study periods. The new classification system is a simple, & reasonable including a detailed description of all the pathologic changes of the joint. The nonsurgical treatment protocol was Simple, effective and specific depending on the pathological changes in joint.

## Introduction

Temporomandibular joint internal derangement (TMJID) is considered the most common Temporomandibular joint (TMJ) disorder^1^. Most studies of TMJID have focused mainly on the position of the disk relative to the condyle and its anterior displacement^2,3^. In 1978, Wilkes used clinical symptoms and surgical and radiological findings, and these were later combined with magnetic resonance imaging (MRI) findings of the TMJ to define the criteria of the TMJID^4,5^.

Previously published studies indicated the association of joint pain and dysfunctional symptoms in cases with disk displacement^6–9^. Furthermore, the results of these studies showed that both disc deformity^10,11^ and bone degenerative changes in hard tissues in the TMJ^12–14^ are other findings associated with TMJ disk displacement.

In Wilkes’ criteria, TMJID was considered a progressing disease. In his radiological stages, Wilkes described disorders starting from symptom-free normal joints associated with slight forward displacement to progressive cases associated with bone degenerative changes with severe clinical symptoms^5^. Other studies have shown that conservative or surgical treatment can result in complete relief of clinical symptoms, although severely displaced disks and severe bone degenerative changes of the joint still exist^15–18^. Moreover, in cases of severely displaced TMJ disks, the clinical symptoms disappeared without any intervention^19–21^. On the other hand, some cases are associated with the clinical signs and symptoms of joint dysfunction and at the same time no disk displacement^22^. Based on the previously mentioned studies, TMJ disc displacements cannot always clarify the changes in clinical symptoms. Focusing only on disk displacement for the diagnosis and treatment of TMJ problems is not sufficient.

The normal function of the TMJ involves complex biomechanics that are multifactorial. One of the factors considered to be important in both normal TMJ biomechanics and TMJ disorders is the muscles acting on the TMJ^23–25^.

Different radiographic imaging techniques have been proposed for evaluating the TMJ, but MRI is still the gold standard imaging modality for the TMJ^26^. A false-positive imaging diagnosis or false-negative imaging diagnosis is suspected if the clinical signs and symptoms do not correlate with the radiographic findings in an MRI study of the TMJ^27^.

Many studies have proposed muscles as one of the major causes of TMJ pain^25,28–30^. An evaluation of pathological changes in the muscles has been missing during the evaluation of temporomandibular joint disorder patients. The benefit of MRI is its ability to evaluate not only the disk-condyle relationship but also the pathological changes of the associated muscles^31–35^.

Although Wilkes provided a staging criterion for TMJID, it did not take into account the state of the lateral pterygoid muscle (LPM), joint effusion, degenerative disc changes, translation of the condyle, or integrity retrodiscal layers (pseudodisk). In addition, the Wilkes classification concentrated on anterior disk displacement and did not mention posterior disk displacement or its associated pathological changes within the joint. Moreover, Wilkes did not suggest a treatment protocol for patients with different stages.

Therefore, the aim of the current study was to establish a new classification of TMJID that can cover all the pathological changes associated with the internal derangement of the TMJ. The new classification system aim to include the state of LPM, joint effusion, degenerative disk changes, osteoarthritic changes of the condyle, translation of the condyle, integrity retrodiscal layers (pseudo-disk) and direction of disk displacement (anterior/posterior). Beside the study aim to propose a nonsurgical treatment protocol based on the MRI finding per each stage of the new classification system.

## Patients and methods

### Ethics statement

The study followed all the tenets of the Declaration of Helsinki for research involving human subjects and was reviewed and approved by the institutional reviewer board of Al-Azhar University School of Dentistry. Informed written consent was obtained from all patients enrolled in the study.

## Patients and study design

### Classification strategy and Patients’ assignment to the stages

To establish the new classification system and evaluate its validity, another independent Retrospective cohort study was conducted for validation of the new classification system. The records of 50 patients including the MRI were evaluated and collected. The primary MRI key points used in the new classification system were**,** absence or presence of the disk displacement, and the direction of the disk displacement (anterior vs posterior). The secondary MRI key point was the pathologic changes within the TMJ.

**Figure 1:**
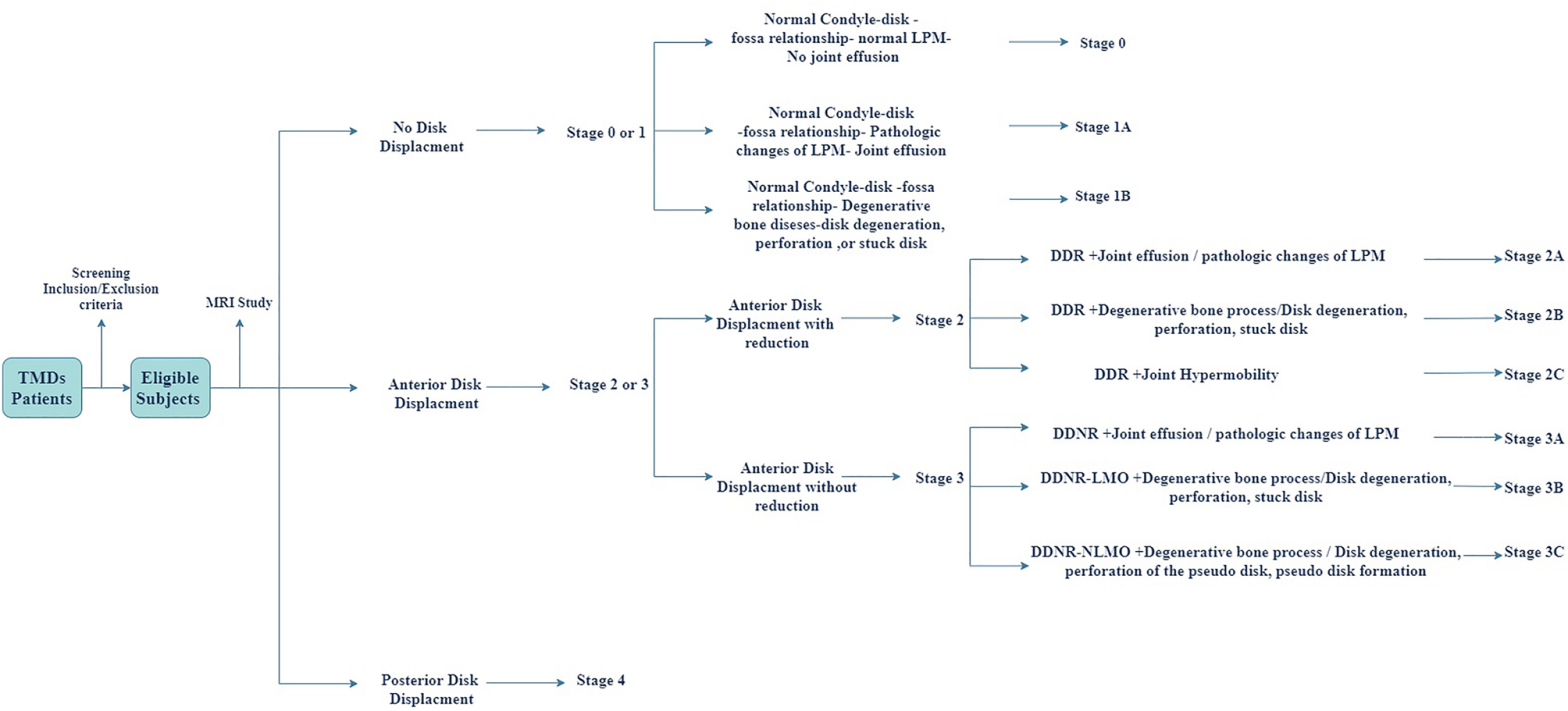
Flowchart represent the process of patients assignment into a group.

The primary key points were used for the classification staging while the secondary key point was used for the sub-staging (Fig. 1). Each MRI was evaluated by our radiologists independently. To validate the new classification system, each radiologist had assigned independently the patients into stage and sub-stage. The cases had assigned into the correct classification stage by both radiologists. The purpose of sub-staging is to detect the degree of the pathological changes within the TMJ with subsequent guidance to a reasonable treatment.

#### The new classification system

Primarily based on MRI, internal derangement of the TMJ is divided into 5 stages.

**Figure 2:**
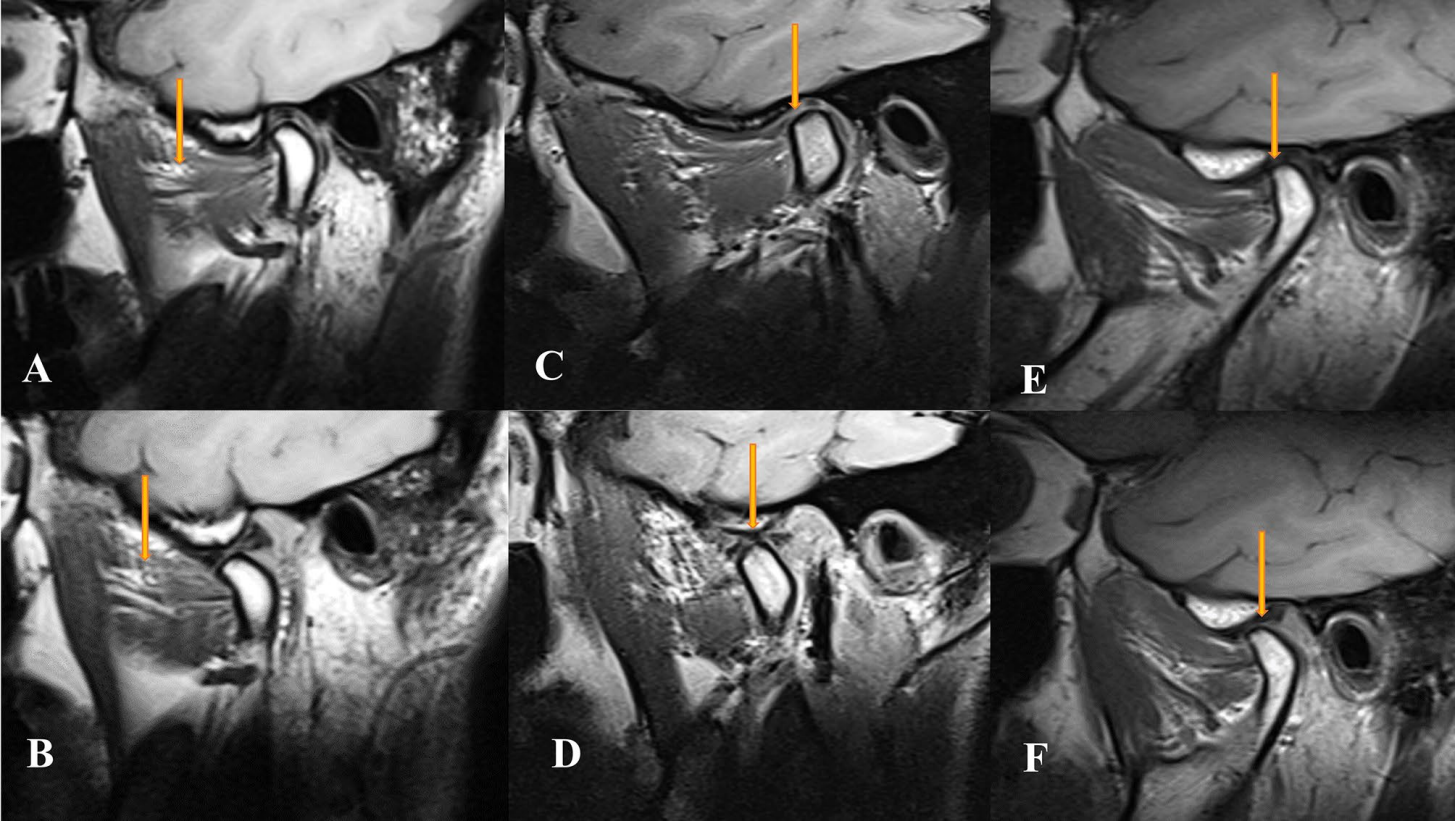
Oblique sagittal T1-weighted images represent stage 1A, with normal Condyle -disk -Fossa relationship in close (**A**) and open (**B**) mouth position with fatty degeneration of the SLPM. Oblique sagittal T1-weighted images represent stage 1B, with normal Condyle -disk -Fossa relationship associated with disk degeneration in close (**C**) and disk perforation in open (**D**) mouth position (the arrow). Oblique sagittal T1-weighted images represent stage IB with normal Condyle -disk -Fossa relationship in close (**E**) and open (**F**) mouth position associated with stuck disk phenomena (the arrow).

**Figure 3:**
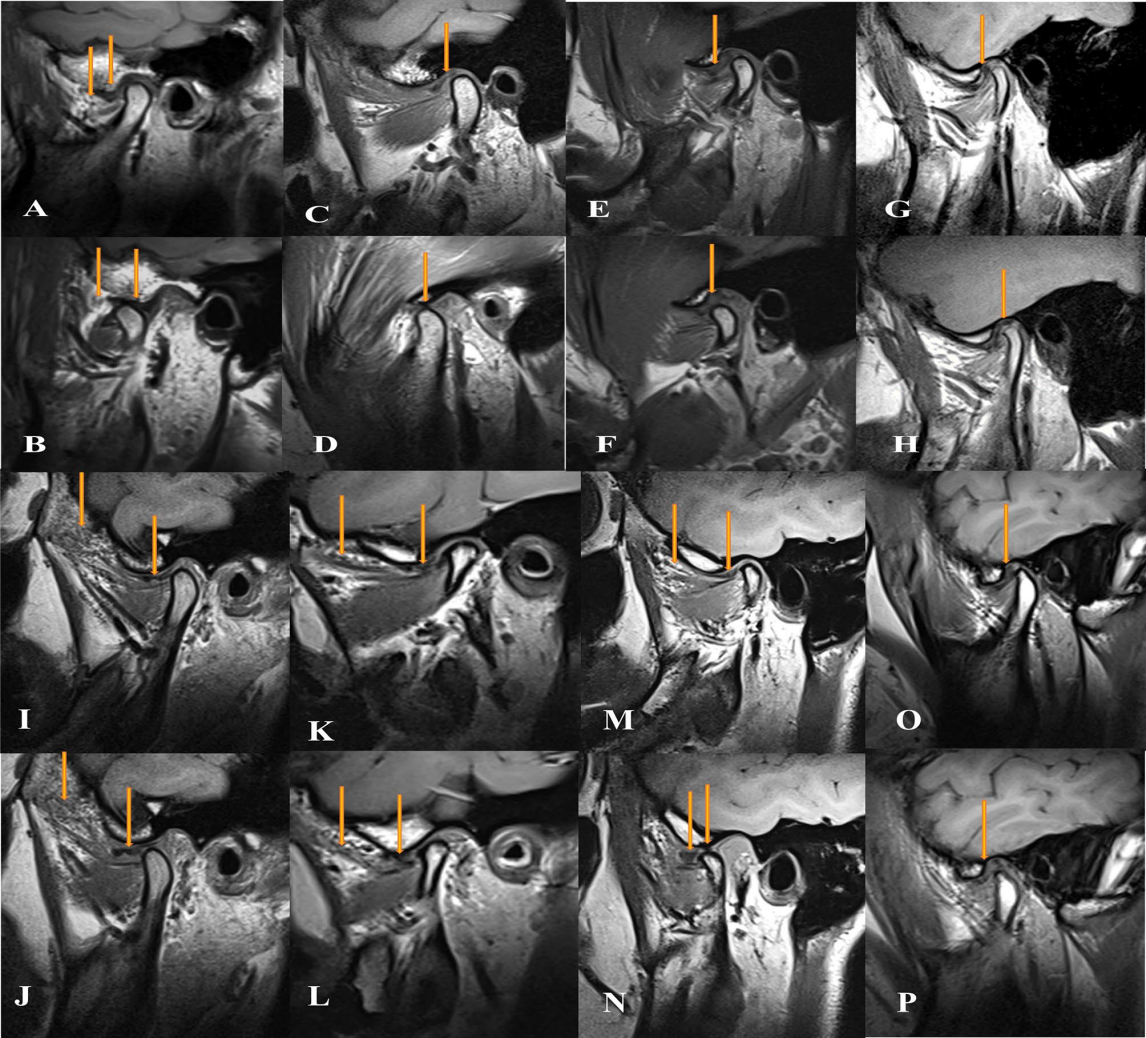
Oblique sagittal T1-weighted images represent stage 2A in close (**A**) and open (**B**) mouth position, DDR associated with fatty degeneration of the Superior head of lateral pterygoid muscle (SLPM) (the arrow). Oblique sagittal T1-weighted images represent stage 2B in close (**C**) and open (**D**) mouth position, with Disk displacement with reduction (DDR) with disk perforation (the arrow). Oblique sagittal T1-weighted images represent stage 2B in close (**E**) and open (**F**) mouth position with DDR associated with stuck disk phenomena (the arrow). Pre-treatment (**G**) oblique sagittal T1-weighted images represent stage 2B, DDR associated with fatty degeneration of the SLPM, disk degeneration, and osteoarthritis of the condyle & Post-treatment (**H**) oblique sagittal T1-weighted images showed disk recapture and condylar remodeling after using the non-surgical treatment protocol. Oblique sagittal T1-weighted images represent stage 3A in close (**I**) and open (**J**) mouth position, Disk displacement without reduction (DDNR) associated with fatty degeneration of the SLPM (the arrow). Oblique sagittal T1-weighted images represent stage 3B in close (**K**) and open (**L**) mouth position, with DDR with disk degeneration (the arrow) and sever osteoarthritic changes of the condyle. Oblique sagittal T1-weighted images represent stage 3C in close (**M**) and open (**N**) mouth position with DDNR associated with fatty degeneration of the SLPM (the arrow), disk degeneration (the arrow), arthritis of the condyle and perforation of the posterior attachment (the arrow in open mouth position). Pre-treatment (**O**) oblique sagittal T1-weighted images represent stage 3C, DDNR associated with Stuck Disk & Post-treatment (**P**) oblique sagittal T1-weighted images showed disk recapture after using the non-surgical treatment protocol.

**Figure 4:**
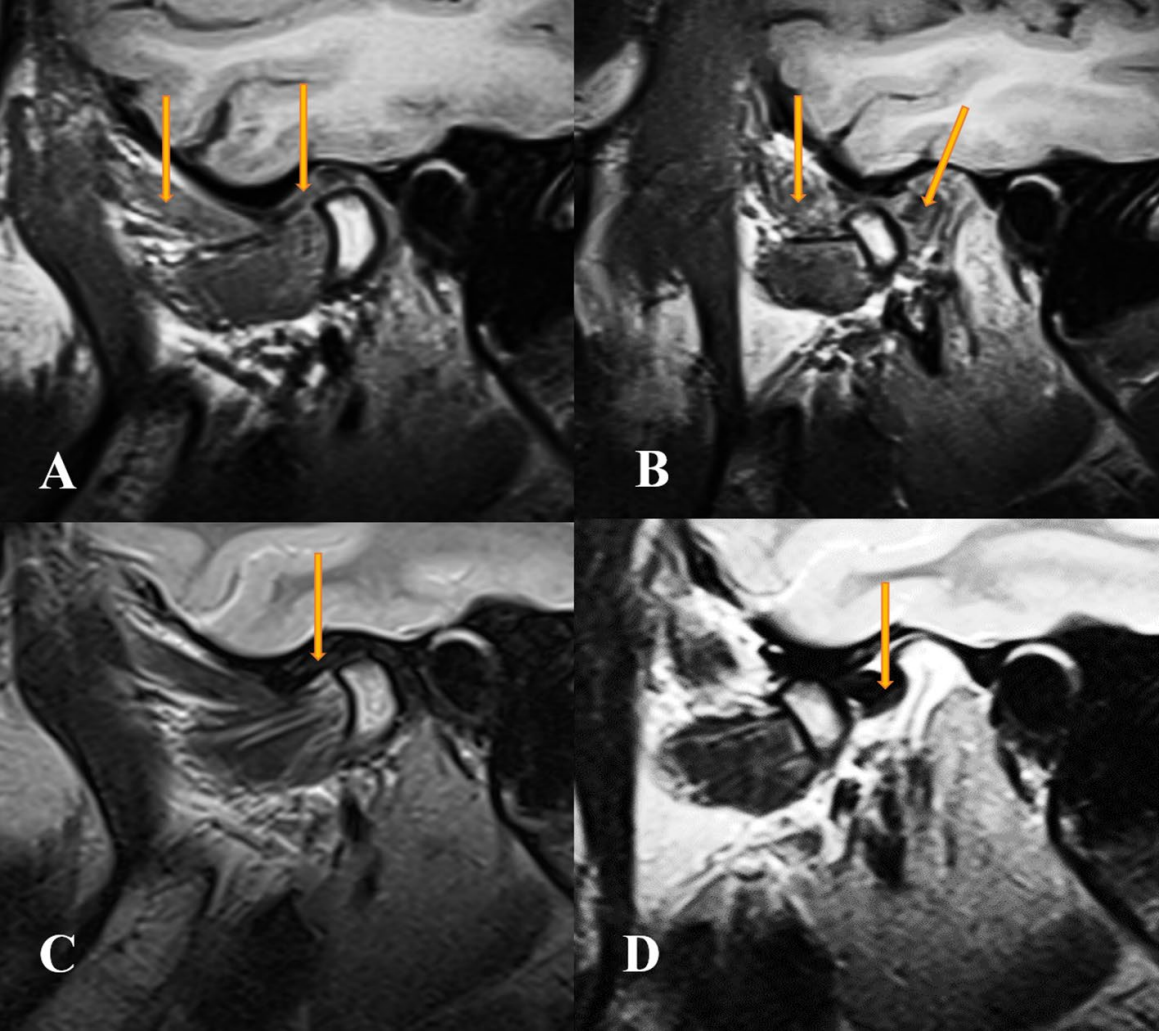
Oblique sagittal T1-weighted images showed normal Condyle -disk -Fossa relationship associated with fatty degeneration of the SLPM and degeneration of the disk in close (**A**) & mouth position with posterior disk displacement of the disk in open (**B**) mouth position (the arrow). Oblique sagittal T2-weighted images showed normal Condyle -disk -Fossa relationship in close (**C**) mouth position with posterior disk displacement of the disk in open (**D**) mouth position (the arrow).

- **Stage 0:** Normal MRI study
- **Stage 1A**: MRI shows a normal condyle-disk-fossa relationship associated with pathologic changes of the LPM + /joint effusion. **Stage 1B**: MRI shows a normal condyle-disk-fossa relationship associated with pathologic changes of the disk + /Bone degenerative process of the condyle (Fig. 2).
- **Stage 2A**: MRI shows anterior disk displacement in the closed mouth position with reduction to the normal position in the open mouth position associated with pathologic changes of the LPM + /joint effusion. **Stage 2B**: MRI shows anterior disk displacement in the closed mouth position with reduction to the normal position in the open mouth position associated with pathologic changes of the disk + /Bone degenerative process of the condyle. **Stage 2C**: MRI shows anterior disk displacement in the closed mouth position with reduction to the normal position in the open mouth position with condylar hypertranslation (Fig. 3).
- **Stage 3A:** MRI shows anterior disk displacement in the closed mouth position without reduction to the normal position in the open mouth position associated with pathologic changes of the LPM + /joint effusion**. Stage 3B:** MRI shows anterior disk displacement in the closed mouth position without reduction to the normal position in the open mouth position associated with pathologic changes of the disk + /Bone degenerative process of the condyle. **Stage 3C:** MRI shows anterior disk displacement in the closed mouth position without reduction to the normal position in the open mouth position associated with normal translation movement of the mandibular condyle (no limitation of the mouth opening) (Fig. 3).
- **Stage 4**: MRI shows posterior disk displacement (Fig. 4).

This prospective clinical cohort study included patients seeking treatment for TMD et al.-Azhar University Hospital and the outpatient clinic at the Faculty of Dental Medicine of Al-Azahr University. The sample originally included patients who provided consent to participate in this study and who ultimately underwent treatment for TMJ Disorders between 2015 and 2017. In the current study, primarily based on MRI, we first developed a new diagnostic classification system, and according to our new classification system, we established therapeutic guidelines.

#### Inclusion criteria

Patients were included if they were older than 18 years and diagnosed with TMJ Disorders. **Exclusion criteria** for this study included systemic diseases (the presence of polyarthritis or other rheumatic diseases), contraindications for MRI (e.g., implanted metal or medical devices, claustrophobia), the presence of neurologic disorders, head and neck cancer, oral submucous fibrosis, a history of TMJ surgery, a history of previous nonsurgical treatment such as occlusal splints, and a history of joint injection with Hyaluronic acid (HA)/platelets rich plasma (PRP). Completely edentulous patients were also excluded. Trauma patients with subcondylar fracture and patients with congenital and developmental disorders of the TMJ were also excluded from the study.

#### Magnetic resonance imaging

MRI scans were examined by 2 radiologists with more than 12 years of experience with MRI of TMJ disorders. Interobserver reliability/consistency (95% confidence interval) for MRI examinations was 0.89 (0.82–0.91), with an excellent level of correlation among the observers. Neither was given any information about the patients, and the MRI images were independently evaluated for the presence or absence of disk displacement, disk degeneration, pathologic changes of the lateral pterygoid muscle, condylar bony changes as signs of bone degenerative process, posterior attachment, or disk perforation. The normal disk position in the sagittal oblique plane was defined as the posterior band of the disk being at the 12 o’clock position relative to the mandibular condyle in the closed mouth position, while in the open mouth position, the thin intermediate band was placed between the mandibular condyle and the articular eminence. Disk displacement with reduction (DDR) in the closed mouth position: the posterior band of the disk is anterior to the condyle, while in the open mouth position, it returns to the normal position between the condyle and articular eminence. Disk displacement without reduction (DDNR): in the closed mouth position, the posterior band of the disk is anterior to the condyle, while in the open mouth position, the posterior band of the disk stays anterior to the condyle and cannot return to its normal position in between the condyle and articular eminence^36,37^. Joint effusion was identified as an area of high signal intensity in the upper or lower joint space on T2-weighted images. Bone degenerative changes of the condyle were defined as the presence of flattening, irregular surface, erosion, osteophytes, and subcondylar cysts. The posterior attachment was evaluated for the presence of perforation, pseudodisk formation, and thinning. Hypertrophy of the LPM manifested as enlargement or an increase in the size of the middle part of the belly. Contracture of the LPM presented as an increase in the size associated with fibrosis manifested as low signal intensity on T2-weighted images. Atrophy of the LPM presented as fatty degeneration with high signal intensity in T1-weighted images^38^.

#### Pretreatment MRI

In our study, we used the MRI imaging protocol established by Hegab et al.^44^. MRI examinations were performed using a 1.5 T unit (Magnetom Vision; Siemens, Erlangen, Germany) with a dual TMJ surface coil. A multislice examination was performed on each patient with 9 slices for each joint in multiple planes (slice thickness 2.5 mm). All patients had bilateral oblique sagittal T1-weighted spin echo scans (repetition time [TR] = 550 ms; echo time [TE] = 13 ms; field of view [FOV] 14 × 14 cm) in both open- and closed-mouth positions. The other available images for review included T2-weighted spin echo images in oblique sagittal (TR = 3570; TE = 67) and closed and open-mouth positions. Proton density–weighted images with spin echo sequences (TR = 3570; TE = 22) were obtained in the oblique sagittal plane in both closed and open-mouth positions. T1-weighted oblique coronal images were acquired in the closed-mouth position only (TR = 550 ms; TE = 13 ms). To prepare for closed-mouth MRI, the clinician instructed the participants to keep the posterior teeth together in a position where the teeth fit the best. The clinician then visually verified this position. The same instructions were reviewed by the radiology technologist, who read them to the patient before the MRI. During open mouth MRI, the clinician instructed the participants to open their mouths as wide as determined previously in the clinic. The patients were instructed to open their mouths as wide as they could without discomfort. The clinician then placed a mouth-opening device between the patient’s teeth and opened the mouth to the maximum that the participant could tolerate. The amount of opening was recorded by the clinician, and this information was given to the radiology technologist. Clinical examinations and MRI studies were used to establish the new classification system.

In the current study, after screening the patients with exclusion and inclusion criteria, the eligible subjects were examined by the MRI. The absence or presence of the disk displacement and its direction (anterior vs posterior) was the first MRI criteria to enrol the patient into a specific stage. Each patient had been assigned to a sub-stage based on MRI evaluation of the associated pathologic changes within the TMJ which Includes the following: pathologic changes of the lateral pterygoid muscles (LPM), joint effusion, Bone degenerative process of the mandibular condyle, pathologic changes of the disk, and condylar hypertranslation.

### Correlation between MRI and clinical finding

To Improve the effectiveness and usefulness of the new classification system, we compared radiological findings with clinical findings. The analysed parameters included, disk position, bone degenerative process, disk perforation, disk degeneration, stuck disk, joint effusion, and pathological changes of the LPM in relation to the mouth opening, pain, and joint sound. To make an objective determination of statistically significant correlations of the MRI and Clinical Findings, the Spearman correlation coefficient was determined.

### Non-surgical treatment protocol therapeutic tools

#### TMJ splint therapy (Hegab TMJ splint)

The main nonsurgical treatment tool. The splint is a hard full coverage maxillary occlusal splint with indentation. The splint was used by the patient all the time except during eating. The splint leads to adjustments of occlusion, enhancement of jaw muscle function, and new positioning of the disk–condyle relationship. The splint vertical thickness was 4-mm vertical splint thickness for DDR and 6-mm vertical splint thickness for DDNR cases and at least 1 year of treatment^44^.

**Functional modification** includes the following: habit awareness, avoid object biting, avoidance of wide yawning, avoid contact sports.

**Pharmacotherapy** (chondroitin sulfate and glucosamine for 12 months)^41^.

#### TMJ arthrocentesis with joint injection

A-PRP injection in cases of Bone degenerative process^42^ and HA injection in cases of stuck disks or degeneration^43^.

**Autologous blood injection**^45^ in cases of joint hypertranslation in combination with Hegab TMJ splints for cases of internal defragment associated with hypermobility.

### Treatment allocation to the different groups

The first and main non-surgical treatment modality is Hegab TMJ splint (HTS) which used in all the classification stages and substages for many reasons, in addition to its placebo effect; it leads to adjustments of occlusion, enhancement of jaw muscle function, and new positioning of the disk– condyle relationship.

The functional modification was used for all the classification stages and substages because it’s aimed to decrease the joint load by decreasing the unnecessary and traumatic joint movements. While Physiotherapy used in TMDs cases associated with muscles disorders.

Pharmacotherapy (glucosamine and chondroitin sulfate) used in substages associated with mild bone degenerative changes.

TMJ arthrocentesis indicated in substages associated with joint effusion to flush the joint and as preparatory step in cases of joint injection. Injection of HA used in substages associated with stuck disk and disk degeneration to improve joint lubrication and disk movement. While PRP injection used in cases of moderate and sever degenerative bone process. The application of blood injection indicated in cases of joint hypermobility.

Table 1 shows a detailed description of the new classification stages associated with the nonsurgical treatment protocol used in our study.

**Table 1:** Detailed description of clinical and MRI finding of the new classification system and treatment protocol for each stage.

Stage	Key findings	Clinical finding	MRI finding	Treatment
Stage 0		1-Normal mandibular range of motions 2-No joint pain 3-No joint sound	1-Normal Condyle-Disk-fossa relationship 2-Normal Lateral Pterygoid muscle (LPM) 3-No Joint effusion	1-No treatment Required
Stage 1: Normal condyle-disk-fossa relationship in close and open mouth position	Stage 1A 1-Functional incoordination 2-Pathologic changes of the LPM	1-Sporadic painless clicking sound 2-Mainly NO joint pain 3-Joint pain in case of joint effusion 4-Normal mandibular range of motions	1-Normal Condyle-Disk-fossa relationship 2-Normal/Pathological changes in LPM Hypertrophy, fatty degeneration, contraction (mainly fatty degeneration) 3-Joint effusion	1-Functional modification 2-Hegab TMJ Splint (HTS-3 mm for 3 months)^39,40^ 3-physiotherapy for the muscles of mastication
	Stage 1B 1-Bone degenerative process 2-Disk Degeneration 3-Disk Perforation 4-Stuck disk	1-Sporadic painless clicking sound 2-Sporadic Catching during mouth opening 3-Joint pain 4-limited mouth opening	1-Normal Disk-condyle-fossa relationship 2-Normal/Pathological changes in LPM Hypertrophy, fatty degeneration, contraction (mainly fatty degeneration) 3-With/without Joint effusion 4-Bone degenerative process 5-Disk perforation 6-Disk degeneration 7-Stuck Disk	1-Hegab TMJ Splint (HTS-3 mm for at least 1 year)-the main treatment modality^39,40^ 2-Functional modification 3-pharmacotherapy (chondroitin sulfate and glucosamine for 12 months)^41^ 4-TMJ arthrocentesis with joint injection A-Platelets rich plasma (PRP) Injection in case of Bone degenerative process^42^ B-Hyaluronic acid (HA) injection in case of stuck disk or degeneration^43^
Stage 2: Anterior disk displacement in closed mouth position with reduction to normal position in the open mouth position (DDR)	Stage 2A 1-Joint effusion 2-Pathologic changes of the LPM	1-No pain in most of the cases 2-Pain in case of joint effusion/posterior position of the condyle 3-Clicking sound with mandibular movements 4-Deviation in mouth opening	1-Anterior disk displacement in closed mouth position with reduction to normal position in the open mouth position 2-With/without Joint effusion 3-Normal /Pathological changes in LPM (Mainly fatty degeneration of LPM)	1-Hegab TMJ Splint Therapy (HTS-4MM for at least 1 year)-the main treatment modality^44^ 2-Functional modification 3-physiotherapy for the muscles of mastication
	Stage 2B 1-Bone degenerative process 2-Disk Degeneration 3-Disk Perforation 4-Stuck disk	1-Joint Pain 2-Clicking sound with mandibular movements 3-crepation 4-Joint tenderness to palpation 4-Deviation in mouth opening	1-Anterior disk displacement in closed mouth position with reduction to normal position in the open mouth position 2-With/without Joint effusion 3-Normal /Pathological changes in LPM (Mainly fatty degeneration of LPM) 4-Bone degenerative process 5-Disk perforation 6-Disk degeneration 7-Stuck disk	1-TMJ Splint (HTS-4 mm for at least 1 year)-the main treatment modality^44^ 2-Functional modification 3-pharmacotherapy (chondroitin sulfate and glucosamine for 12 months)^41^ 4-TMJ arthrocentesis with joint injection A-PRP Injection in case of Bone degenerative process^42^ B-HA injection in case of stuck disk or degeneration^43^
	Stage 2C 1-TMJ hypermobility 2-Disk Displacement with reduction (DDR) 3-clicking sound	1-Pain/ no pain 2-Clicking/ no clicking sound 3-No limitation of the mouth opening 4-Joint Hypertranslation	1-Anterior disk displacement in closed mouth position with reduction to normal position in the open mouth position 2-condylar Hypertranslation 3-with/without Joint effusion 4-Normal /Pathological changes in LPM (Hypertrophy or atrophy or fatty degeneration mostly Fatty degeneration)	1-TMJ Splint (HTS-4 mm for 6 months)^44^ followed by TMJ arthrocentesis & blood injection^45^ with splint in situ 2-Functional modification
Stage 3: Anterior disk displacement in closed mouth position without reduction to normal position in the open mouth position (DDNR)	Stage 3A 1-Joint effusion 2-Pathologic changes of the LPM	1-History of clicking sound 2-Limited mouth opening 3-Contralateral excursion less than 7 mm 4-Uncorrected deflection to the affected side on opening (unilateral case) 5-No deflection with mouth opening in bilateral cases 6-Joint Pain in case of joint effusion or posterior position of the condyle	1-Anterior disk displacement in closed mouth position without reduction to normal position in the open mouth position 2-with/without Joint effusion 3-Normal /Pathological changes in LPM (Hypertrophy or fatty degeneration—mostly fatty degeneration)	1-TMJ Splint Therapy (HTS-6MM for at least 1 year)-the main treatment modality^44^ 2-Functional modification 3-TMJ arthrocentesis with joint injection (HA)^43^ 4-pharmacotherapy (chondroitin sulfate and glucosamine for 12 months)^41^ 5-physiotherapy for the muscles of mastication
	Stage 3B 1-Disk Displacement without reduction with limited mouth opening (DDNR-LMO) 2-Bone degenerative process 3-Disk Degeneration 4-Stuck disk	1-History of clicking sound 2-Limited mouth opening 3-Contralateral excursion less than 7 mm 4-Uncorrected deflection to the affected side on opening (unilateral case) 5-No deflection with mouth opening in bilateral cases 6-Joint Pain 7-crepatus	1-Anterior disk displacement in closed mouth position without reduction to normal position in the open mouth position 2-with/without Joint effusion 3-Normal /Pathological changes in LPM (Hypertrophy or atrophy or fatty degeneration—mostly fatty degeneration of SLPM) 4-Bone degenerative process 5-Disk degeneration 6-Stuck disk	1-TMJ Splint Therapy (HTS-6MM for at least 1 year)- the main treatment modality^44^ 2-Functional modification 3-pharmacotherapy (chondroitin sulfate and glucosamine for 12 months)^41^ 4-TMJ arthrocentesis with joint injection A-PRP Injection in case of Bone degenerative process^42^ B-HA injection in case of stuck disk or degeneration^43^
	Stage 3C 1-Disk Displacement without reduction without limited mouth opening (DDNR-NLMO) 2-Psudeo disk formation 3-perfortaion of the pseudo disk 4-Bone degenerative process 5-Disk degeneration	1-History of clicking sound 2- on-Limited mouth opening 3-No deflection with mouth opening 4-Joint pain mostly due to arthritis	1-Anterior disk displacement in closed mouth position without reduction to normal position in the open mouth position 2-with/without Joint effusion 3-Normal /Pathological changes in LPM (Hypertrophy or atrophy or fatty degeneration—mostly fatty degeneration of SLPM) 4-Bone degenerative process 5-Disk degeneration 6-Pseudo Disk Formation 7-Perforation of pseudo disk	1-TMJ Splint Therapy (HTS-6MM for at least 1 year)-the main treatment modality^44^ 2-Functional modification 3-pharmacotherapy (chondroitin sulfate and glucosamine for 12 months)^41^ 4-TMJ arthrocentesis with joint injection A-PRP Injection in case of Bone degenerative process^42^ B-HA injection in case of stuck disk or degeneration^43^
Stage 4: Posterior disk displacement	1-Posterior disk displacement 2-Severe Bone degenerative process 3-Disk perforation 4-Disk degeneration	1-Joint pain 2-limited mouth opening 3-Deviation in mouth opening	1-Normal disk position in close mouth with posterior disk displacement in open mouth position 2-Posterior disk displacement in close mouth position with reduction into normal in open mouth position 3-Posterior disk displacement in close mouth without reduction in open mouth position 4-Severe Bone degenerative process 5-Disk perforation 6-Disk degeneration	1-TMJ Splint Therapy (HTS-6MM for at least 1 year)-the main treatment modality^44^ 2-TMJ arthrocentesis / joint injection (PRP)^42^ 3-Functional modification 4-pharmacotherapy (chondroitin sulfate and glucosamine for 12 months)^41^

Classification of temporomandibular joint internal derangement.

TMJ: Temporomandibular joint, LPM: Lateral Pterygoid muscle, HTS: Hegab Temporomandibular joint splint, PRP: platelets rich plasma, HA: Hyaluronic acid.

### Evaluating the outcomes of the treatment protocol

**The primary outcome variable** was treatment effectiveness based on the assessment of pretreatment and posttreatment maximum nonassisted (voluntary) mouth opening (**MVMO**) in millimetres. Pretreatment and posttreatment pain index scores were measured using a 10-point visual analogue scale (**VAS**), with 0 indicating absence of pain and 10 indicating the worst pain^44^. **The secondary outcome variable** was **joint sound**^44^. To evaluate pretreatment and posttreatment joint sound, the patients were asked to open their mouths as widely as possible, and the joint sound was then determined by combining 3 means: (1) palpation of the TMJ zone by the clinician, (2) the patient’s self-reporting regarding whether the joint sound could be heard, and (3) auscultation of the TMJ zone with the stethoscope. The absence of joint sound was confirmed when no sound was detected or reported when the above 3 means were employed. Joint sound was considered to be present if it was detected/reported with use of the above 3 means or a result was undetermined.

All outcome variables were assessed and compared within the groups at baseline pretreatment and posttreatment 1, 3, 6, and 12 months later. Age and gender were considered the third category of variables and correlated with the outcome variables. Adjustment variables included baseline MVMO and the pain index score. For statistical purposes, VAS pain levels and jaw range-of-motion values were managed as continuous variables. For all variables, repeated measures analysis of variance was performed to assess the existence of significant within-group and between-group treatment effects. Adjustments for age, gender and affected sides (unilateral/bilateral) were performed to assess the influence of demographic features on treatment effectiveness^44^.

### Statistical analysis

A post hoc power analysis was designed to determine the study’s power. The power was found to be 0.97 (97%), indicating that the sample size was adequate. The sample size calculation was performed using G*Power version 3.1.9.2. The numerical data were explored for normality by examining the distributions of the data, calculating the means and medians, and using tests of normality (e.g., the Kolmogorov–Smirnov and Shapiro–Wilk tests). The age data exhibited a parametric distribution, but the inter-incisal opening data exhibited a nonparametric distribution. The VAS scores were also treated as nonparametric data. The age data, presented as the mean and standard deviation (SD) values, were compared using Student’s t test. The nonparametric data are presented as median and range values. The Mann–Whitney U test was used for between-group comparisons. The Friedman test was used to study the changes in each group over time. Wilcoxon signed rank tests were used for pairwise comparisons when Friedman tests yielded significance. The joint sound data (qualitative data) are presented as frequencies (n) and percentages (%). The significance level was set at 0.05. The data were analysed using InStat statistical software (GraphPad Software, Inc., La Jolla, CA)^44^.

## Results

### The results of the retrospective Cohort study

Interobserver reliability/consistency (95% confidence interval) scores between the radiologists for MRI examinations was 0.91 (0.87–0.93), with an excellent level of correlation among the observers. All the 50 cases had assigned into the correct classification stage and substage. The primary and secondary key points in the sagittal view used to enrol the patients into the stage and substage made the process simple and accurate. The additional axial view evaluation did not statistically change the agreement among the radiologists. The validation of the new classification system showed an overall almost perfect agreement by both radiologists.

### Patient demographic data

During the study interval, 500 patients were screened for eligibility. Thirteen patients did not meet the inclusion criteria (11 had rheumatoid arthritis, and 2 had psoriasis). Four patients could not undergo MRI because of claustrophobia and were excluded from the study. Fifteen patients had previous TMJ treatment (occlusal splint, joint injection with HA/PRP). Two patients had a history of trauma and subcondylar fracture. Thirty-one patients were unwilling to receive any treatment after clinical and MRI evaluation, and they were excluded from the study. The final sample was composed of 435 patients (292 female and 143 male) with a total number of 747 joints. Out of the total patients, 308 were bilateral cases (210 F & 98 M), and 127 were unilateral cases (82 F & 45 M). The patient age ranged from 19 to 57 years, with a mean age of 34.35 ± 8.2. Table 2 shows the descriptive statistics of the patients enrolled in the study.

**Table 2:** Demographic features and baseline values in outcome variables within each of study group.

Study Group	1A	1B	2A	2B	2C	3A	3B	3C	4
Sample size	26	186	20	61	9	6	101	27	15
**Gender**									
Male	11	60	5	22	3	3	32	17	9
Female	15	126	15	39	6	3	69	10	6
Mean age	38.46 ± 6.2	36.7 ± 8.6	28.1 ± 5.9	32.97 ± 8.8	34.6 ± 5.4	36.2 ± 6.3	31.6 ± 5.6	35.4 ± 6.7	46.6 ± 8.7
Mouth Opening	35.92 ± 1.4	36.3 ± 2.1	38.0 ± 1.1	37.9 ± 2.2	46.2 ± 1.7	29.7 ± 3.4	29.6 ± 2.1	38.1 ± 1.3	39.19 ± 3.1
Pain (VAS)	3.7 ± 0.7	4.6 ± 1.2	5.8 ± 1.14	6.7 ± 0.9	7.7 ± 0.7	8.0 ± 0.9	7.4 ± 0.9	7.2 ± 1.1	8.25 ± 0.2
Joint Sound	0.23 ± 0.4	0.5 ± 0.04	1.0 ± 0.0	09.5 ± 0.22	1.0 ± 0.0	1.7 ± 0.4	0.35 ± 0.48	0.37 ± 0.09	0.5 ± 0.52

### MRI findings

Table 3 shows a detailed description of the MRI findings per study.

**Table 3:** Detailed description of the MRI finding in all of the study groups.

	Total joint numbers	LPM pathology	Joint effusion	Arthritis	Disk degeneration	Disk perforation	Stuck disk	Pseudo disk formation	Pseudo disk perforation	Hypertranslation	Posterior disk displacement
Group 1A	37	13 (35%)	5 (14%)	0	0	0	0	0	0	0	0
Group 1B	287	101 (35%)	20 (7%)	138 (48%)	203 (70%)	53 (18%)	18 (6%)	0	0	23 (8%)	0
Group 2A	34	7 (21%)	15 (44%)	0	0	0	0	0	0	0	0
Group 2B	103	12 (12%)	13 (12%)	71 (69%)	56 (54%)	20 (19%)	6 (6%)	0	0	14 (13%)	0
Group 2C	18	10 (56%)	0	14 (78%)	16 (89%)	2 (11%)	0	0	0	18 (100%)	0
Group 3A	12	12 (100%)	8 (67%)	0	0	0	0	4 (33%)	0	0	0
Group 3B	202	76 (38%)	34 (17%)	192 (95%)	176 (87%)	0	22 (11%)	67 (335)	47 (22%)	0	0
Group 3C	54	12 (22%)	6 (1.8%)	48 (89%)	48 (89%)	0	2	38 (70%)	6 (1.8%)	0	0
Group 4	24	16 (67%)	0	24 (100%)	24 (100%)	11 (46%)	0	0	0	5 (21%)	24 (100%)
Percentage per total number of joints	747	259(34.7%)	101(13%)	487(65%)	523(70%)	86(11%)	96(13%)	109(14%)	53(7%)	60(8%)	24(3%)

An MRI study of the patients enrolled in the current study at 12 months posttreatment without splints in the mouth showed disk recapture in 19 cases of DDR and 9 cases of DDNR (stage 3A & 3B). None of the cases of stage 3C showed disk recapture. And No Joint effusion were found at the end of the treatment.

### Results of the correlation between MRI and clinical finding

To correlate the radiological findings with clinical findings, Therefore, each Spearman-Rho coefficient was calculated to make an exact statement. Statistical analysis showed highly significant positive correlation between the MVMO (Increase mouth opening) and DDR, Disk Degeeneration, Pseudo disk formation (*P* < 0.0001).

While there was a highly significant negative correlation between the MVMO (decrease mouth opening) and DDNR, Bone degenerative process, joint effusion, stuck disk (*P* < 0.0001). significant negative corelation between MVMO and LPM pathology and disk perforation (*P* = 0.0317 & 0.007 respectively).

Statistical analysis showed highly significant positive correlation between the VAS (increase VAS) with DDR, DDNR, posterior disk displacement, and bone degenerative process (*p* < 0.0001). Joint effusion, Disk degeneration, and LPM pathological changes showed significant positive correlation to VAS (*P* = 0.008, 0.003, & 0.04 respectively). While stuck disk & pseudo disk formation showed non-significant correlation to VAS (*P* = 0.999 & 0.0512 respectively).

Statistical analysis showed high significant correlation between the Joint sound with DDR and disk perforation (*P* =  < 0.0001 & 0.043 respectively). While all the other MRI finding showed non-significant correlation to Joint sounds (*P* > 0.05).

### Clinical outcome results of the treatment protocol

Maximum nonassisted (voluntary) mouth opening **(MVMO):** Statistical analysis of the differences between pretreatment and posttreatment measurements showed an increase in mouth opening throughout the study period, with a statistically significant increase at 12 months posttreatment in study groups 1, 2, &3 (*P* < 0.001). Statistical analysis of the mouth opening in Group 4 at 12 months posttreatment showed a nonsignificant increase in the mouth opening. (*P* = 0.1242).

The pain index score **(VAS):** Statistical analysis of the **VAS** showed a statistically significant decrease in the pain score in all study groups during all study periods. *P* < 0.0001 at 12 months posttreatment in all study groups.

**Joint Sound:** Statistical analysis of the **joint sound** showed significant improvement of the joint sounds with all groups **1A, 1B, 2A, 2B, 2C, 3B, & 3C** during all study periods. Only **group 3A** showed nonsignificant improvement in the joint sound (*P* = 0.2936).

Table 4 shows the clinical characteristics of the different pretreatment and posttreatment changes of the clinical outcome variables in all study groups.

**Table 4:** clinical characteristics of the different pre-treatment and post-treatment changes of the clinical outcome variable in all the study groups.

Variables groups	MVMO		VAS		Joint sounds	
	Pre-treatment	12 Months post-treatment	Pre-treatment	12 Months post-treatment	Pre-treatment	12 Months post-treatment
Group 1A	35.92 ± 0.26	40.23 ± 0.27	3.6 ± 0.17	0.0	0.23 ± 0.08	0.0
*P* value	< 0.0001***		< 0.0001***		0.043*	
F value	13.31		2.012		2.190	
Df	4		4		4	
Coefficients	0.3080		0.03178		0.3067	
Group 1B	36.58 ± 0.15	38.57 ± 0.30	4.6 ± 0.09	0.0	0.35 ± 0.03	0.0
*P* value	< 0.0001***		< 0.0001***		< 0.0001***	
F value	3.261		2.565		2.560	
Df	4		4		4	
Coefficients	0.4149		0.06062		0.3231	
Group 2A	36.7 ± 0.25	39.75 ± 0.22	5.9 ± 0.25	0.0	0.95 ± 0.05	0.05 ± 0.05
*P* value	< 0.0001***		< 0.0001***		< 0.0001***	
F value	4.579		2.574		3.156	
df	4		4		4	
Coefficients	0.2408		0.02949		0.2190	
Group 2B	37.87 ± 0.29	40.39 ± 0.15	6.7 ± 0.12	0.0	0.95 ± 0.028	0.0
*P* value	< 0.0001***		< 0.0001***		< 0.0001***	
F value	12.81		3.551		2.634	
Df	4		4		4	
Coefficients	0.5375		0.03033		0.2071	
Group 2C	46.33 ± 0.39	39.89 ± 0.33	7.9 ± 0.18	0.0	0.94 ± 0.055	0.11 ± 0.08
*P* value	< 0.0001***		< 0.0001***		< 0.0001***	
F value	336.0		4.208		2.526	
df	4		4		4	
Coefficients	0.9781		0.02347		0.1992	
Group 3A	29.67 ± 0.93	39.17 ± 0.32	8.0 ± 0.25	0.0	0.25 ± 0.13	0.08 ± 0.08
*P* value	< 0.0001***		< 0.0001***		0.2936^ ns^	
F value	5.281		4.750		1.238	
df	4		4		4	
Coefficients	0.1079		0.01724		0.1477	
Group 3B	29.14 ± 0.16	39.49 ± 0.07	7.5 ± 0.06	0.0	0.36 ± 0.034	0.005 ± 0.005
*P* value	< 0.0001***		< 0.0001***		< 0.0001***	
F value	3.409		2.883		2.215	
df	4		4		4	
Coefficients	0.1257		0.01821		0.2881	
Group 3C	38.45 ± 0.17	39.62 ± 0.18	5.9 ± 0.23	0.018 ± 0.019	0.3585 ± 0.07	0.0
*P* value	< 0.0001***		< 0.0001***		< 0.0001***	
F value	12.59		3.052		2.095	
df	4		4		4	
Coefficients	0.6727		0.07659		0.2712	
Group 4	39.19 ± 0.78	40.63 ± 0.46	8.2 ± 0.23	0.06 ± 0.06	0.50 ± 0.13	0.06 ± 0.06
*P* value	0.1242^ ns^		< 0.0001***		0.005**	
F value	36.59		3.261		2.071	
df	4		4		4	
Coefficients	0.8491		0.01719		0.23200.2320	

MVMO: Maximum voluntary mouth opening, VAS: Visual analogue scale.

*P* value > 0.05 non-significant.

*P* value < 0.05 significant.

df: Degree of Freedom.

## Discussion

The final sample was composed of 435 patients (292 female and 143 male) with a total number of 747 joints. At the end of the study; disk recapture in 19 cases of DDR and 9 cases of DDNR (stage 3A & 3B). None of the cases of stage 3C showed disk recapture. And No Joint effusion were found at the end of the treatment. Statistical analysis of the of the clinical outcome variables post-treatment showed significant increase in the mouth opening and significant decrease in the pain score in all study groups.

Reasons for classification: Classification is considered the process of transforming descriptions of the diagnosis of pathologic findings into universal medical codes that represent the data required for evidence-based treatment plans. Missing data will result in insufficient treatment plans with failure to achieve excellent outcomes and relapse. Staging is a measure of disease severity based solely on predefined medical criteria. Classification staging makes it replicable, easy to audit, and applicable. During the course of TMJ diseases, there are discrete "stages" that are manifested and can be defined and detected by the MRI, reflecting the severity of the disease. These stages have clinical significance for prognosis and therapeutic modality.

The main problem regarding the classification systems of TMJ internal derangement is lack of understanding of the meaning of the internal derangement. This is why classifications vary within the literature with some focus on disc/condyle relationships, while others extend to include Joint Bone degenerative process changes. Moreover, some classifications extend to include muscle disorders, while others completely pull apart the muscle diagnosis with TMJ internal derangement.

Wilkes^5^ considered internal derangement a progressive disorder starting from stage I to stage V. In our study, we found patients with disk perforation without disk displacement, which is contrary to the rationale of Wilkes’ classification.

The most recent classification of internal derangement was based on the scoring of MRI abnormalities (100 patients) without clinical correlation and without a proposed treatment protocol^58^.

In our study, we found cases with a normal disk condyle relationship that were at the same time associated with disk perforation; such cases would be stage I or stage V Wilkes classification. What type of treatment is indicated in such cases? Are they considered normal cases or diseased cases? Does the patients need any intervention or just follow-up? How do the cases have disk perforation without passing through Wilkes stages I to V? What about posterior disk displacement cases and their treatment?

The Wilkes classification^5^ is the most widely used classification and has been adopted by surgeons who treat TMJ disorders. Its widespread adoption is linked to its simplicity in describing escalating joint pathology in 5 stages as a progressive disorder, but it concentrates on only 2 disorders (internal derangement and Bone degenerative process) and fails to include other TMJ disorders, such as pathologic changes in the LPM, joint effusion, and state of posterior attachment. In addition, Wilkes did not propose a treatment protocol, and its predictive value for the TMJ is still unclear.

In our classification system, we covered all aspects of the pathologic joint conditions that can be evaluated by MRI with the proposed nonsurgical treatment protocol for every stage in the classification system. Our classification is mainly dependent on MRI because it is the gold standard for the TMJ, and MRI is currently regarded as the standard criterion for the evaluation of TMJ internal derangements. MRI is capable of providing information on disk morphology and position through high soft-tissue resolution without exposing the patient to ionizing radiation. It is a simple and non-invasive procedure and is now widely used worldwide. Beside it include the posterior disk displacement which not included in the previous classifications of TMJ internal derangement. Moreover, the new classification system benefits in guiding the non-surgical treatment plan with predicable prognosis.

To our knowledge, our staging system is the first classification system to demonstrate the state of LPM, joint effusion, degenerative disc changes, disk perforation, Bone degenerative process of the condyle, translation of the condyle, and the integrity of retrodiscal layers (pseudodisk) in addition to the direction of disk displacement (anterior/posterior) contributing to a nonsurgical treatment protocol. Our work demonstrated that, by following the process of the classification, the patients can easily be assigned into the right stage and substage.

It was believed that degeneration and perforation of the disc appears to occur secondary to disc displacement as disease progresses, which is the rationale of the Wilkes classification^46^. With respect to the pathological variation, the results of the Wilkes study showed that 97% of patients presented intermediate, intermediate-late, or late stage status^5^. In our study, we found disk degeneration and disk perforation, which were, according to the Wilkes stages, of intermediate-late or late stage status in joints with a normal disk condyle relationship (stage 1B- 287 joints represent 38% of total joints). This means that the disease is not a progressive disorder, as supposed by Wilkes, and the exact cause of the internal derangement of the TMJ depends on all factors, including the biomechanics of the joint, not only the disk and condyle.

A decrease in signal intensity on T1-weighted MR images has been described in the posterior attachment in chronically displaced discs, thought to represent the formation of a “pseudo” disc with fibrotic remodelling of the posterior attachment in the region of its attachment to the posterior band of the disc^47^. This is most commonly seen in chronically displaced discs, which is in agreement with our results, which represent 20% of the joints in stage 3 (DDNR).

TMD is multifactorial in origin, which is why many theories try to explain the complex aetiology of the disorder. The Pathologic changes in the lateral pterygoid muscle are considered by some authors to be among the possible factors that can lead to TMD. Furthermore, in an attempt to explain the causative factor of the TMD, some authors considered uncoordinated function between the upper and lower heads of the LPM to be the factor responsible for abnormal disk position^49^, and some studies concluded that contraction of the muscle attached to the articular disk is the cause of disk displacement^50^. However, the exact roles of function and dysfunction of the LPM in disk displacement are still unclear^51,52^.

Excessive overloading of skeletal muscles has been reported to be an important factor leading to muscle hypertrophy with secondary changes in the muscle, producing atrophy or contracture of the muscle^38^.

The rationale behind the role of LPM in disk displacement varies among studies. Some authors believe muscle hyperactivity is the causative factor, while other studies have shown that hypoactivity is the causative factor. Moreover, the variable pattern of muscle attachment has been considered by other authors to be the causative factor of TMJ internal derangement^53–57^. In our study, the main pathologic change of the LPM was fatty degeneration (atrophy) of the superior head of the LPM. Hypertrophy and contraction were present in a few cases.

The effectiveness of our nonsurgical treatment protocol in reducing joint pain was statistically significant in all of the study groups. Statistical analysis of the **VAS** showed a statistically significant decrease in the pain score in all study groups during all study periods, with *P* < 0.0001 at 12 months posttreatment in all study groups.

Regarding the improvement of the MVMO, statistical analysis of the differences between pretreatment and posttreatment measurements showed an increase in mouth opening throughout the study period, with a statistically significant increase at 12 months posttreatment in study groups 1, 2, and 3 (*P* < 0.001). Statistical analysis of the mouth opening in Group 4 at 12 months posttreatment showed a nonsignificant increase in the mouth opening (*P* = 0.1242). Our nonsurgical treatment protocol was effective in all study groups, except in stages 3C and 4 because these two stages were not associated with limited mouth opening.

In stage 3C, DDNR without limited mouth opening can be explained by disc deformation; the posterior disc attachment appears stretched and becomes increasingly thinner and, in some cases, becomes remodelled and thicker and acts as a pseudodisk. This partially explains the increasing opening capacity of the jaw that many patients experience after initially suffering from limitation of opening with disc displacement without reduction. Loosening or tearing of the joint capsule and detachment or perforation of the posterior disc attachment are probably responsible for the increased jaw mobility that frequently occurs over time. In this group of patients, the main complaint was pain that resulted from associated joint Bone degenerative process. In our study, 89% of joints with stage 3C disease were associated with joint Bone degenerative process and disk degeneration.

**Joint Sound:** Statistical analysis of the **joint sound** showed significant improvement of the joint sounds with all groups **1A, 1B, 2A, 2B, 2C, 3B, & 3C** during all study periods. Only **group 3A** showed nonsignificant improvement in the joint sound (*P* = 0.2936).

The findings of our study suggest that the nonsurgical treatment protocol is associated with improvement of the clinical outcomes in all study groups.

In our study, 19 cases of DDR and 9 cases of DDNR (stage 3A & 3B) had disk recapture, while none of the cases of stage 3C showed disk recapture. This is because in stage 3C, the posterior attachment changed to a fibrotic band and acted as a pseudodisk. In such cases, the posterior attachment cannot regain its normal form and subsequently its normal function. Disk recapture is a complex process depending on many factors, such as the direction of disk displacement, degree of disk displacement, integrity of posterior attachments, shape and integrity of the disk, degree of condylar degeneration and arthritic changes, amount of joint load, and harmony between the superior and inferior heads of the lateral pterygoid muscles. In general terms, disk recapture depends on factors related to normal biomechanics of the TMJ.

Application of splint thickness of 4 mm and 6 mm for cases of DDR and DDNR, respectively, resulted in anteroposterior and vertical movements of the mandibular condyle with anteroposterior movements of the articular disk, which play an important role in disk recapture, as confirmed by the MRI study^44^.

The application of arthrocentesis and injection of HA in cases of stuck disks help to improve the lubrication system of the joint with subsequent free disk movement^43^. In cases of joint Bone degenerative process, the application of arthrocentesis and injection of PRP have been proven to have better performance than HA in terms of joint pain reduction and increased MVMO^42^.

The application of blood injection in the treatment of mandibular hypermobility is considered a simple and safe technique and can be used in outpatient clinics^45^. In our study, we used the splint first for stage 2C to ensure reestablishment of a good disk-condyle relationship before application of blood injection, which will lead to fibrosis of the joint capsule and will render disk recapture or reestablishment of a good disk condyle relationship. In addition, the presence of the splint inside the mouth associated with movements of the condyle and the disk resulted in repositioning of the disk over the condyle. Therefore, in our study, we performed blood injection with the splint in situ (splint inside the mouth).

One of the limitations of our study is the need to enrol a large number of patients in some of the stages (such as stage 2C & 3A), and this could not be overcome in our study, as we try to establish a new classification system with new classification criteria.

Another limitation of our study is lack of the control group to compare the results of our non-surgical treatment protocol. This is because it’s difficult to choose a single treatment modality from the wide variety of the non-surgical treatment methods in the literature as a control. Beside our treatment protocol include a combination of many treatment modalities depending on the MRI findings in each stage. This study was limited by being a monocenter study, clinical and MRI findings were compared in only symptomatic group.

## Conclusion

The new classification system is reasonable, reliable, and feasible system including a detailed description of all the pathologic changes of the joint. The nonsurgical treatment protocol was Simple & effective and specific depending on the pathological changes in joint. Moreover, the new classification system benefits in guiding the non-surgical treatment plan with predicable prognosis.
